# Pseudomyogenic hemangioendothelioma/epithelioid sarcoma-like hemangioendothelioma of the lower limb: report of a rare case

**DOI:** 10.1186/s13000-015-0384-z

**Published:** 2015-08-28

**Authors:** Chuifeng Fan, Lianhe Yang, Xuyong Lin, Enhua Wang

**Affiliations:** Department of Pathology, First Affiliated Hospital and College of Basic Medical Sciences of China Medical University, 110001 Shenyang, China; Institute of Pathology and Pathophysiology, China Medical University, 110001 Shenyang, China

## Abstract

Pseudomyogenic hemangioendothelioma is an extremely rare soft tissue tumor, also named as epithelioid sarcoma-like hemangioendothelioma, which occurs more frequently in young adult males. It was originally recognized as a variant of epitheloid sarcoma, however it is now concluded as a distinctive, rarely metastasizing endothelial neoplasm. We present a case of pseudomyogenic hemangioendothelioma in the lower limb in a 49-year-old female who has a long course of disease and suffered from twice local recurrences and lymph node affection of the tumor. The mass was subcutaneous and the margins were ill-defined. Morphologically, the tumor cells show diversity, composed of large spindle cells and round cells, both with abundant eocinophilic cytoplasm, mimicking rhybdomyoplasts and epitheloid cells respectively. The tumor cells show diffuse strong expression of Factor VIII, Fli-1, INI-1, vimentin, MDM2, and CDK4, local expression of CD31, AE1/AE3, EMA and P63, and no expression of CD34, S-100, actin-sm, desmin, MyoD1, and HMB45. Based on these information, this case is diagnosed as pseudomyogenic hemangioendothelioma after ruling out the main differential diagnosises including epithelioid sarcoma, malignant peripheral nerve sheath tumor and rhabdomyosarcoma. From this case we suggest that pseudomyogenic hemangioendothelioma may be confused with a variety of soft tissue neoplasm histologically. The clinical feature of the case of a long course of disease with twice local recurrences and final lymph node involvement 10 years after excision of the primary tumor indicates a relative indolent behavior of this tumor.

## Background

Pseudomyogenic hemangioendothelioma, also known as epithelioid sarcoma-like hemangioendothelioma, occurs more frequently in young adult males and usually arises in the extremities, especially on the lower limb, and often involves multiple tissue planes [1–5]. It histologically mimics a myoid tumor or epithelioid carcinoma due to abundant eosinophilic cytoplasm and the cell shape [1–5]. It is now characterized as a distinctive, rarely metastasizing endothelial neoplasm [1]. The tumor is composed of large round or spindle cells, or commonly both parts, arranged in sheets or fascicles. The cells rarely show notable pleomorphism or nuclear atypia [1–3]. Clinically, more than half of the patients experience local recurrence, and lymph node and distant metastasis in a few cases has also been reported [1–9]. In the case we present here, the patient experienced a long course of disease and twice recurrences and lymph node involvement of the tumor after excision of the primary tumor 10 years ago. Morphologically, the tumor is composed of both areas of round and spindle cells with abundant eocinophilic cytoplasm, which may lead to confusion with a variety of other soft tissue neoplasm such as epithelioid sarcoma, malignant peripheral nerve sheath tumor and rhabdomyosarcoma. The immunophenotype of expression of INI-1, AE1/AE3, and endothelial markers such as Factor VIII, Fli-1 and CD31, and negative staining of desmin, MyoD1, and S-100 supports the diagnosis of pseudomyogenic hemangioendothelioma in this case.

## Case presentation

### Clinical history

A 49-year-old female referred to our hospital for recurrence of soft tissue tumor with multiple nodules in her left lower limb. MRI shows that the nodules were subcutaneous at the left calf below the knee and integrated to a mass about 6 cm × 5 cm. The mass has been growing up gradually for 2 years without treatment. Before that, the patient had a primary tumor about 1 cm × 1 cm in the same location about 10 years ago. The patient experienced once recurrence with a new mass at the same location around the incision 2 years after local excision of the primary one. This new mass grew up to about 5 cm × 3 cm in 3 years and then the patient received a second surgery. And now it is the second recurrence of the tumor and the third time for her to receive surgery. MRI also detected a subcutaneous mass about 3.2 cm × 3.4 cm at her left thigh near the inguinal area this time. Blood test of the patient shows no abnomity. The urinary test shows slight high levels of red blood cells (5.11/HPF), white blood cells (3.09/HPF), epithelial cells (2.02/HPF) and urea (7.73 mmol/L).

### Materials and methods

The resected specimens of the tumor were fixed with 10 % neutralbuffered formalin and embedded in paraffin blocks. Tissue blocks were cut into 4 μm-thick sections and the sections were dewaxed in xylene and rehydrated stepwise in descending ethanol series. Then the sections were boiled in citrate buffer (pH 6.0) within an autoclave. Endogenous peroxidase activity and non-specific binding were blocked with 3 % H_2_O_2_ and non-immune sera, respectively. The sections were then incubated with the following primary antibodies: actin-sm (1:50, DAKO), AE1/AE3 (1:50, DAKO), CD31 (1:100, DAKO), CD34 (1:100, DAKO), cytokeratin 18 (CK18, 1:200, DAKO), cytokeratin 19 (CK19, 1:200, DAKO), CDK4 (1:50, Abcam), desmin (1:50, DAKO), EMA (1:100, DAKO), Fli-1 (1:100, Abcam), Factor VIII (1:100, Abcam), HMB45 (1:50, Abcam), INI-1 (1:100, Santa Cruz), Ki67 (1:200, DAKO), Melan-A (1:50, DAKO), myoD1 (1:50, DAKO), MDM2 (1:50, Abcam), P63 (1:100, DAKO), S-100 (1:50, DAKO), and vimentin (1:200, DAKO) overnight at 4 °C. Thereafter, the catalyzed signal amplification system (Maixin Biotechnology, Fuzhou, Fujian, China) was used for staining of these proteins according to the manufacturer’s instructions. The antibodies were detected by a standard avidin-biotin complex method with biotinylated secondary antibodies (Maixin) and an avidin-biotin complex (Maixin), and developed with diaminobenzidine. Counterstaining was done lightly with hematoxylin, and the sections were dehydrated in alcohol before mounting. This study was prospectively performed and approved by the institutional Ethics Committees of China Medical University and conducted in accordance with the ethical guidelines of the Declaration of Helsinki.

## Results

### Gross features

**Fig. 1:**
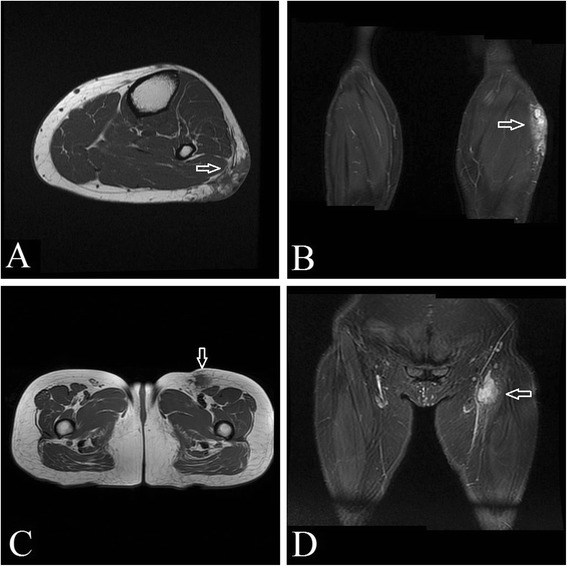
The imaging (MRI) of the tumor. MRI shows multiple subcutaneous nodules at the left calf below the knee integrating to a mass about 6 cm × 5 cm. The biggest one was about 1.4 cm × 0.9 cm. (**a**, T1-weighted image shows low signal intensity, ; **b**, T2-weighted image (STIR) shows high signal intensity, ). MRI also detected a subcutaneous mass about 3.2 cm × 3.4 cm at the left thigh near the inguinal area (**c**, T1-weighted image shows low signal intensity, ; **d**, T2-weighted image (STIR) shows high signal intensity, )

The resected samples inspected include nodules from the left calf and thigh of the patient. The masses are subcutaneous with multiple nodules integrated with each other and the margins are ill-defined. The mass from the calf is about 5.5 cm × 4.2 cm × 2.5 cm, and the one from the thigh is about 3.0 cm × 3.0 cm × 2.5 cm. The cut surface of the masses is firm, and grey and yellowish white. MRI shows multiple subcutaneous nodules at the left calf below the knee integrating to a mass about 6 cm × 5 cm. The biggest one was about 1.4 cm × 0.9 cm (Fig. 1a, T1-weighted image shows low signal intensity; 1B, T2-weighted image (STIR) shows high signal intensity). MRI also detected a subcutaneous mass about 3.2 cm × 3.4 cm at the left thigh near the inguinal area (Fig. 1c, T1-weighted image shows low signal intensity; 1D, T2-weighted image (STIR) shows high signal intensity).

### Microscopic features

**Fig. 2:**
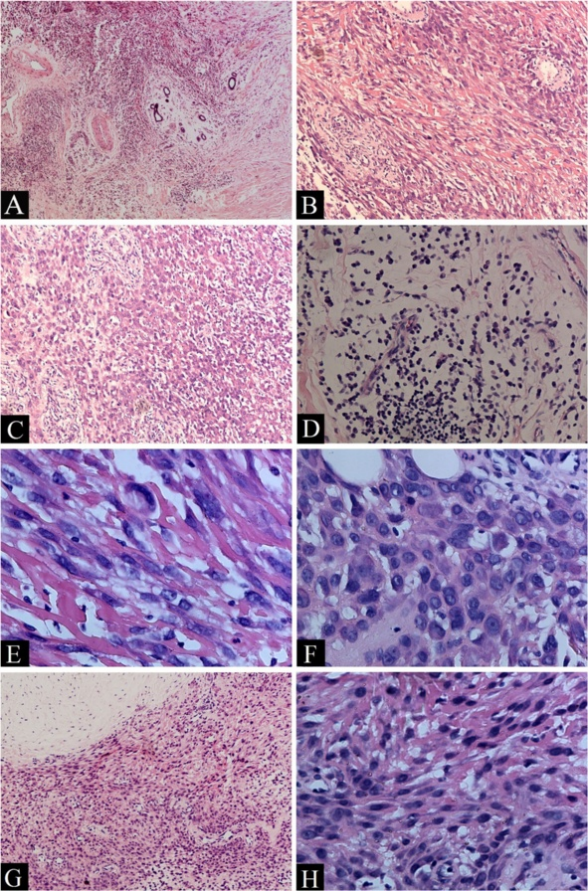
Morphological features of the tumor. Ill-defined tumor cells are diffusely distributed between the subcutaneous tissues (**a**,×100). Most of the tumor tissues are cellular with large plump spindle cells (**b**,×200) or round cells (**c**,×200). Some areas of the tumor have myxoid stroma and scattered small round tumor cells (**d**,×200). The tumor cells are spindle (**e**,×100) or round (**f**,×400) in different areas, both with abundant eocinophilic cytoplasm. The tumor cells are large and plump, showing no apparent pleomorphism. The nuclei of the tumor cells have small nucleoli without notable atypia and the mitotic activity is scarce (**e**, **f**,×400). The microscopic features of the primary tumor were the same as the recurrent tumor (**g**, ×100; **h**,×400)

The tumor cells are diffusely distributed between the subcutaneous tissues with an invasive growth pattern and ill-defined (Fig. 1a). Most of the tumor tissues are cellular, and a few areas of the tumor have myxoid stroma and scattered tumor cells (Fig. 1b, c, d). The tumor cells are round or spindle in different areas, both with abundant eocinophilic cytoplasm, arranged in sheets, fascicles or irregularly (Fig. 1b, c, d, e, f). The tumor cells are large and plump, but show no apparent pleomorphism. The nuclei of the cells are also plump with small nucleoli without notable atypia and the mitotic activity is scarce (Fig. 1e, f). The microscopic features of the primary tumor were the same as the recurrent tumor (Fig. 1g, h). The microscopic feature of the mass from the left thigh indicates lymph node metastasis of the tumor (Fig. 2).

### Immunophenotype

**Fig. 3:**
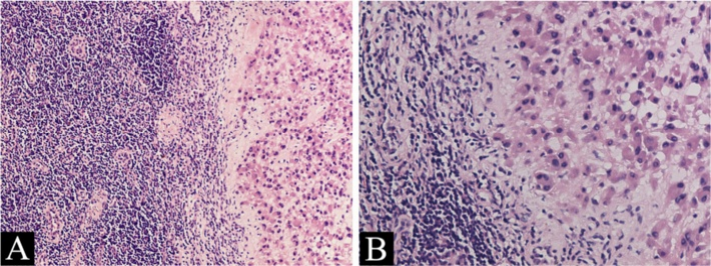
Lymph node metastasis of the tumor in the thigh far from the primary lesion of the recurrenct tumor and the original tumor. The large round epithelioid tumor cells are similar in shape to those in the tumor from the lower limb of the patient (**a** × 100; **b** × 200)

Immunohistochemical examination indicates that the tumor cells were diffusely positive for Factor VIII, Fli-1, CDK4, INI-1, MDM2, vimentin, focally positive for AE1/AE3, CD31 and negative for actin-sm, CD34, desmin, myoD1 and S-100 (Fig. 3). Ki67 index was about 5 % (Fig. 3). In addition, EMA and P63 expression was focal, CK18, CK19, HMB45 and melan-A were negative in the tumor cells. The primary tumor also shows a typical Fli-1 and INI-1 positive immunostaining phenotype (Fig. 5).

**Fig. 4:**
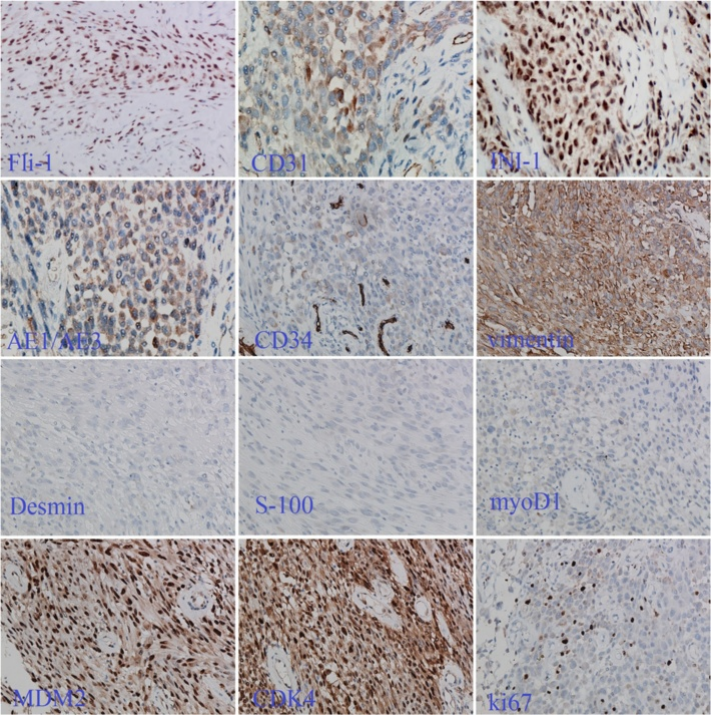
Immunohistochemical staining of the tumor. Fli-1 in tumor cells was diffusely positive and the staining was strong as well as in the endothelial cells. CD31 was focally positive. INI-1 expression was retained. AE1/AE3 staining was focal and not very strong. CD34 in tumor cells was very weak or absent compared to endothelial cells. Vimentin was strongly and diffusely positive. Desmin, S-100 and myoD1 were negative. MDM2 and CDK4 were strongly and diffusely positive. Ki67 index was about 5 %

**Fig. 5:**
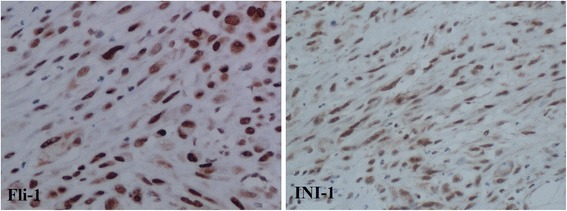
Immunohistochemical staining of the primary tumor. It also shows a typical immunostaining phenotype of positive Fli-1 and INI-1 expression as the recurrent tumor

## Discussion

Pseudomyogenic hemangioendothelioma, also named as epithelioid sarcoma-like hemangioendothelioma, which morphologically mimics a myoid tumor or epithelioid carcinoma, is now concluded as a distinctive, rarely metastasizing endothelial neoplasm [1–5]. This tumor occurs more frequently in young adult males and usually arises in the extremities [1–5]. In the case we presented here, the patient was a 49-year-old female who had a long course of disease about 10 years with this tumor in her lower limb.

The tumor is composed of large spindle cells, arranged in sheets or fascicles. Tumor cells with epithelioid cytomorphology are also often present [1–5]. In this case, the tumor is subcutaneous and both areas with spindle cells and round epithelioid cells exist. The cells are large and have abundant eosinophilic cytoplasm, mimicking a myoid tumor or epithelioid carcinoma cells. The tumor cells are plump, but show no apparent pleomorphism. The nuclei of the cells have small nucleoli without notable atypia and the mitotic activity is scarce. Based on these information, the main differential diagnosises should include epithelioid sarcoma, malignant peripheral nerve sheath tumor and rhabdomyosarcoma. Epithelioid sarcoma is a mesenchymal neoplasm exhibiting epithelioid cytomorphology, the classic subtype of which often occurs in the extremity [1]. Spindle tumor cells can also present in this tumor. The cell pleomorphism and nuclear atypia are not notable usually in this tumor either. However classic epithelioid sarcoma usually consists of cellular node with central degeneration which is not seen in this case though it cannot be ruled out only based on this feature. The tumor cells in this case exhibit abundant eosinophilic cytoplasm mimicking myoid tumor. Thus rhabdomyosarcoma or leiomyosarcoma should also be considered. Leiomyosarcoma is a malignant neoplasm exhibiting pure smooth-muscle differentiation [1]. The tumor consists mainly of spindle sells arranged in fascicles. However epithelioid cytomorphology can also exist, though relatively rare. The nuclear pleomorphism is generally notable in leiomyosarcoma which was not seen in this case. There are several subtypes of rhabdomyosarcoma, among which spindle cell/sclerosing rhabdomyosarcoma is the most important to be differentiated for this case. This subtype has spindle cell morphology with eosinophilic cytoplasm and shows a fascicular or plaxiform growth pattern [1]. Epithelioid cells are rare and nuclear atypia is common in this subtype, which is different from this case. Malignant peripheral nerve sheath tumor is a malignant tumor arising from peripheral nerves, which mostly arises in the extremities [1]. Typical type of this tumor consists of spindle cells showing a fascicular growth pattern. However there is also an epithelioid subtype of this tumor composed of epithelioid cells with abundant eosinophilic cytoplasm, which should also be considered for this case. The neoplasmic cells of this type are cohesive together or scattered and embedded in the extracellular myxoid matrix. The similar areas also exist in this case. However prominent nucleoli are often seen in the tumor cells of this type but not in this case. Moreover areas with spindle cells are also main parts of the tumor in this case which is not usual in the epithelioid subtype of malignant peripheral nerve sheath tumor. However, a variety of complicated and similar histological features can present in all these tumors and the microscopic features could not be the only basis for differentiation for these tumors.

In the current case, immunohistochemical examination shows that the tumor cells were diffusely positive for endothelia cell markers Factor VIII and Fli-1 and focally positive for CD31. However these markers are not absolutely specific for endothelial cells. The other mesenchymal tumors mentioned above should be ruled out. INI-1 expression has been found to be lost in some tumors including epithelioid sarcoma [1]. In this case INI-1 expression was retained. The myoid tumor markers such as actin-sm and desmin were negative in this case. S-100, a marker usually positive in tumors arising from nervous system including malignant peripheral nerve sheath tumor, was also negative in this case. However even in malignant peripheral nerve sheath tumor, S-100 expression is not always detected [1]. We need to differentiate theses tumors more carefully based on more information but not only on one marker like S-100. vimentin was diffusely positive in the tumor cells, which supports but cannot fully prove the tumor as a mesenchymal neoplasm. As for keratins, AE1/AE3 was focally but not diffusely positive in this case. AE1/AE3 was usually positive in cases of pseudomyogenic hemangioendothelioma reported so far [1–5, 10–14]. Some documents mentioned that the immunostaining pattern is diffuse. However the documents are limited and more cases are still in need to be studied to verify the immunostaining pattern of this marker. CD34 expression is usually negative in pseudomyogenic hemangioendothelioma, however a report indicates that it was positive in some cases [3]. In a word, the immunophenotype of this tumor is still to be studied and characterized. From these information it may be more reasonable to considere this case as an endothelial neoplasm but not myoid tumor, peripheral nerve sheath tumor, or epithelioid sarcoma. Ki67 index in this case was about 5 % and not very high. MDM2 and CDK4 overexpression are not usually seen in benign tumors but quite common in intermediate or malignant mesenchymal tumors [15, 16] and was diffusely positive in this case. These three markers are helpful to understand the nature of the tumor and support the consideration of it as a tumor with a certain extent of malignant potentials.

However, the histological feature and immuostaining findings are not enough for fully understanding and characterizing the tumor of this case as usual. The clinical feature is important for the diagnosis and should be carefully studied as always. The patient of this case had experienced a long course of disease with the tumor about 10 years which indicates the tumor is relative indolent. Approximately 60 % of patients with pseudomyogenic hemangioendothelioma experience local recurrence of the tumor [1]. In this case the patient experienced twice local recurrences at the same location after local excisions. After 10 years the tumor has developed a regional subcutaneous lymph node metastasis in her thigh. No distant metastasis in other organs was found in this patient. Pseudomyogenic hemangioendothelioma is now concluded as a intermediate, rarely metastasizing endothelial neoplasm. The clinical feature of this patient is also in accordance with that of pseudomyogenic hemangioendothelioma. Cancer can be caused by various factors including inflammation which can promote the formation of premalignant lesions and promote cancer progression. The immune system plays complicated roles in the progression of malignant tumors [17]. In this case, the patient experienced a couple times of recurrence and at last developed lymph node metastasis. However, there wasn’t any clue of inflammation or auto-immune diseases related found in this patient. So far, the pathogenesis and mechanism involved in the progression of this tumor is not uncovered.

## Conclusion

Pseudomyogenic hemangioendothelioma is a rare endothelial neoplasm which often mimics myoid and epithelioid tumors morphologically. A variety of mesenchymal neoplasms with similar architecture and cell morphology should be carefully differentiated from this tumor. For the diagnosis the immunostaining is very important but not decisive and enough. Clinical information including age, gender, tumor location, disease course and recurrence is important for appropriate diagnosis and fully understanding of the tumor and is indispensable. Analysis based on any single factor or incomplete information may easily lead to arbitrary conclusion. However the reports of this tumor are limited. Further studies on more cases are in need for fully understanding and appropriate diagnosis of it.

## Consent

Written informed consent was obtained from the patient for publication of this case report and accompanying images.
